# mTOR inhibition as a novel gene therapeutic strategy for diabetic retinopathy

**DOI:** 10.1371/journal.pone.0269951

**Published:** 2022-06-16

**Authors:** Steven Hyun Seung Lee, Joo Yong Lee, Jun-Sub Choi, Hee Jong Kim, Jin Kim, Seho Cha, Kyoung Jin Lee, Ha-Na Woo, Keerang Park, Heuiran Lee

**Affiliations:** 1 CdmoGen Co., Ltd., Cheongju, Korea; 2 Department of Ophthalmology, Asan Medical Center, University of Ulsan, College of Medicine, Seoul, Korea; 3 Bio-Medical Institute of Technology, University of Ulsan, College of Medicine, Seoul, Korea; 4 Department of Microbiology, University of Ulsan, College of Medicine, Seoul, Korea; 5 Department of Biochemistry & Molecular Biology, University of Ulsan, College of Medicine, Seoul, Korea; 6 Department of Microbiology, Asan Medical Center, University of Ulsan, College of Medicine, Seoul, Korea; PTC Therapeutics, UNITED STATES

## Abstract

In addition to laser photocoagulation, therapeutic interventions for diabetic retinopathy (DR) have heretofore consisted of anti-VEGF drugs, which, besides drawbacks inherent to the treatments themselves, are limited in scope and may not fully address the condition’s complex pathophysiology. This is because DR is a multifactorial condition, meaning a gene therapy focused on a target with broader effects, such as the mechanistic target of rapamycin (mTOR), may prove to be the solution in overcoming these concerns. Having previously demonstrated the potential of a mTOR-inhibiting shRNA packaged in a recombinant adeno-associated virus to address a variety of angiogenic retinal diseases, here we explore the effects of rAAV2-shmTOR-SD in a streptozotocin-induced diabetic mouse model. Delivered via intravitreal injection, the therapeutic efficacy of the virus vector upon early DR processes was examined. rAAV2-shmTOR-SD effectively transduced mouse retinas and therein downregulated mTOR expression, which was elevated in sham-treated and control shRNA-injected (rAAV2-shCon-SD) control groups. mTOR inhibition additionally led to marked reductions in pericyte loss, acellular capillary formation, vascular permeability, and retinal cell layer thinning, processes that contribute to DR progression. Immunohistochemistry showed that rAAV2-shmTOR-SD decreased ganglion cell loss and pathogenic Müller cell activation and proliferation, while also having anti-apoptotic activity, with these effects suggesting the therapeutic virus vector may be neuroprotective. Taken together, these results build upon our previous work to demonstrate the broad ability of rAAV2-shmTOR-SD to address aspects of DR pathophysiology further evidencing its potential as a human gene therapeutic strategy for DR.

## Introduction

Currently a leading cause of blindness, diabetic retinopathy (DR) is poised to become a greater global health concern, as one quarter [1] of the projected 592 million diabetes mellitus (DM) patients by 2035 [2] are estimated to develop DR to sight-threatening levels. A degenerative condition, DR initially presents as non-proliferative diabetic retinopathy (NPDR) before progressing to proliferative diabetic retinopathy (PDR), with the latter being characterized by neovascularization (NV), a process driven by vascular endothelial growth factor (VEGF) and linked to DR-related vision loss [3]. A complicating variable to the pathophysiology of DR and its treatment options is the concurrent development of diabetic macular edema (DME), which can occur during either NPDR or PDR [2], is marked by macular thickening or swelling [4], and is the most common cause of DR-related blindness [2–4].

As they most directly threaten the vision of DR patients and due to the central role played therein by VEGF, the standard of care for both PDR and DME consists of either anti-VEGF therapeutics delivered via intravitreal injections [2] and/or laser photocoagulation [5]. Multiple safety and efficacy issues exist for the latter [6], including subretinal fibrosis formation [7], vascular leakage, and the observed new development of choroidal NV [6], all contributors to DR progression. On the other hand, protein-based intravitreal anti-VEGF treatments are relatively short lived, requiring frequent administration to adequately suppress VEGF on a long-term basis [2, 5]. This may be burdensome to the patient both procedurally and economically, negatively affecting patient compliance [8], which is problematic due to the progressive [3] and degenerative [5] nature of DR. Significant portions of the patient population have additionally been found to be unresponsive to these therapeutics [3, 4], and safety concerns have been raised regarding long-term VEGF suppression [9, 10], on top of injection-related risks [5].

Meanwhile, DR is a highly multifactorial disorder [3, 5] with a number of processes contributing to its progression, including the systemic effects of diabetes-related hyperglycemia, oxidative stress, inflammation, and hypoxia-induced effects on retinal vessels. These factors largely overlap with those that drive DME development, particularly angiogenesis [11], and Müller cell activity [12]. Therefore, a therapeutic strategy focused on a target with broader effects, such as the mechanistic target of rapamycin (mTOR), may be more efficacious than currently available treatment options in addressing the various aspects of DR and DME progression and pathophysiology. A serine/threonine kinase involved in a number of cellular processes and signaling pathways [13], mTOR has been previously identified as a potential therapeutic target for a number of angiogenic retinal conditions [10], including PDR [14], while mTOR dysfunction has been implicated in various ocular disorders [15].

Additionally, the ability to elicit long-term effects without the need for frequent intravitreal injections would overcome a major limitation of widely-used anti-VEGF drugs, for which gene therapy is particularly well-suited, and we have previously explored the direct inhibition of mTOR via RNA interference (RNAi) as a human gene therapeutic strategy. A novel mTOR-inhibiting short hairpin RNA (shRNA) with broad multi-species activity was designed using a program developed in-house [16], then packaged into a recombinant adeno-associated virus vector (rAAV) [17]. Nonpathogenic in nature and capable of transducing dividing and non-dividing cells to elicit long-term transgene expression, rAAVs are particularly well-suited for delivering gene therapeutics targeting ocular conditions [18]. Next, we examined in a rat model of oxygen-induced retinopathy (OIR) the therapeutic potential of the mTOR-inhibiting virus vector versus various angiogenic retinal disorders, including retinopathy of prematurity and DR [19]. However, the presence of a GFP reporter gene in the rAAV expression cassette and the accompanying cytotoxic and immunogenic effects [20] thereof meant that its direct use as a human therapeutic would be inappropriate. The GFP reporter transgene was then replaced with a stuffer DNA having null expression [21] and the resulting virus vector, rAAV2-shmTOR-SD, was shown to be as effective as its GFP-containing counterpart in its in vivo activity [22].

Here, rAAV2-shmTOR-SD was tested in a STZ-induced diabetic mouse model which recapitulates early DR processes that contribute to overall pathoprogression [23]. Upon delivery via intravitreal injection, it was found to effectively transduce the retina to reduce mTOR expression. Active in a variety of retinal cell types associated with DR pathophysiology, the therapeutic virus vector was able to reduce pericyte loss, the formation of acellular capillaries, leaky vessel development, and the thinning of retinal cell layers, while also having an anti-apoptotic effect. In conjunction with our previous work showing that rAAV2-shmTOR-SD is anti-angiogenic [19], our results here further demonstrate its promise as potential gene therapeutic for angiogenic ocular disorders, DR in particular.

## Materials and methods

### Experimental virus vector development

We have previously described the generation of rAAV2-shmTOR-SD and rAAV2-shCon-SD [19] from pAAV-shmTOR-GFP and pAAV-shCon-GFP, their respective precursor plasmids [24]. Briefly, the mTOR-inhibiting shRNA (5’-GAAUGUUGACCAAUGCUAU-3’) or a control shRNA (5’-AUUCUAUCACUAGCGUGAC-3’) was inserted into a self-complementary AAV2 vector under the control of an H1 promoter alongside a stuffer DNA derived from the human *UBE3A* gene. All virus vectors used in this study were obtained from CdmoGen Co., Ltd. (Cheongju, Korea).

### STZ-induced diabetic mouse model and animal care

A well-established protocol [25] for the induction of diabetes mellitus in mice via a single high-dose of streptozotocin was followed, with minor modifications, using 7-week-old male C57/BL6 mice obtained from The Orient Bio (Sungnam, Korea). All animal care and experiments were conducted in accordance with the Association for Research in Vision and Ophthalmology Resolution on the Use of Animals in Ophthalmic and Vision Research and was overseen by the Institutional Animal Care and Use Committee of the University of Ulsan, College of Medicine at Asan Medical Center (study protocol approval number: 2018-14-082; approval date: May 1, 2018). The Animal Research: Reporting of In Vivo Experiments Guidelines were adhered to as well.

75 animals were used overall in order to overcome any potential deaths during the generation of the diabetic mouse model and ensure that 5 mice were able to be allocated into each of the 4 experimental groups for the 3 separate experimental time periods, to yield a total number of 60 study mice. Mice were housed in a 25° C temperature-controlled room with a photoperiod of 12 hours of light and dark apiece, and free access was provided to water and chow. The cages, housing 5 mice apiece, were kept in the same area of the room, with no other controlling of confounders occurring.

Mice were injected intraperitoneally with 150 mg/kg of streptozotocin (Sigma-Aldrich, St. Louis, MO) instead of the 200 mg/kg specified to mitigate potential death due to STZ-induced toxicity, and 10% sucrose water provided only to mice whose condition deteriorated significantly, determined by reductions in body weight (S1 Table). Tail vein blood samples were taken 1 week post-STZ treatment to select successfully generated diabetic mouse models with blood glucose levels exceeding 300 mg/dL (S2 Table), determined via Accu-Chek (Roche Diagnostics, Indianapolis, IN).

Upon generation of the diabetic mouse model, experimental groups of equal average blood glucose levels were made and the mice randomized via double blind grouping. During the investigation, only the authors who performed animal care and experimentation were aware of the group allocations, with the other authors being made aware during data analysis. Health monitoring for the mice in this study consisted of regular visual inspections, as well as regular blood glucose level and urine volume checks. If a mouse’s condition deteriorated significantly, they were to be sacrificed, but no other efforts were made beyond health monitoring to reduce potential suffering.

### Intravitreal injections and sacrifice

One month post-STZ treatment, mice were anesthetized with a 4:1 mixture of 40 mg/kg Zoletil (zolazepam/tiletamine) from Virbac (Carros Cedex, France) and 5 mg/kg of Rompun (xylazine) from Bayer Healthcare (Leverkusen, Germany) administered intraperitoneally, and their pupils dilated using Mydrin-P (0.5% tropicamide and 2.5% phenylephrine) from Santen (Osaka, Japan). Both eyes were then injected intravitreally with 1 μL of the virus vectors at a concentration of 5.0 x 10^10^ viral genomes (v.g.)/mL. Sacrifice occurred at either 1, 2, or 5 months post-intravitreal injection. A 4:1 mixture of Zoletil (80 mg/kg) and Rompun (10 mg/kg) was used to deeply anesthetize the mice prior to intracardial perfusion with 0.1 M PBS (7.4 pH) containing 150 U/mL heparin and infusion with 4% paraformaldehyde (PFA) in 0.1 M phosphate buffer (PB). Eyecups were generated by enucleating the eyeballs, fixing for 1 hour in 4% PBA in 0.1 M PB, and removing the anterior sections, including the cornea and lens. The experimental unit was considered to be one mouse, for a total of 5 per experimental group and 60 overall for the study.

### Tissue preparation and immunohistochemistry

Eyecups were placed overnight in 30% sucrose in PBS before being embedded in Tissue-Tek (Miles Scientific, Napierville, IL), an optimal cutting temperature compound, and 5–10 μm-thick frozen transverse retinal sections prepared. Section samples were stained with anti-mTOR (AF15371; R&D Systems, Minneapolis, MN) to visualize the in vivo efficacy of the mTOR-inhibiting shRNA, while retinal cells were visualized using anti-NeuN (MAB377; Millipore, Burlington, MA), anti-GFAP (12389; Cell Signaling Technology, Danvers, MA), or anti-GS (MAB302; Millipore). The samples were incubated overnight at 4°C with the diluted primary antibodies, washed 3 times in PBST for 10 minutes apiece, incubated for 2 hours at room temperature with the secondary antibodies Alexa Fluor 568 or 488 (Thermo Fisher Scientific, Waltham, MA), and stained with DAPI (D9542; Sigma-Aldrich). Section samples were examined using a LSM 710 fluorescence confocal microscope (Carl Zeiss Microscopy, Jena, Germany). Images were captured using the black edition of Zeiss Zen software (Carl Zeiss Microscopy) and analyzed using ImageJ (National Institutes of Health, Bethesda, MD). mTOR values were quantified by calculating Alexa 568 fluorescence intensity as a function of area at 200X magnification for 5 randomly selected areas of the retinal section samples and presented as a ratio relative to normal control mice.

### Retinal trypsin digest

After fixing enucleated eyeballs in 10% formalin solution for 24 hours, followed by washing in PBS and isolating the retina, trypsin digestion [26] was performed with minor modifications. The retina was incubated at 37°C with gentle shaking in a 3% trypsin solution (15090046; Thermo Fisher Scientific) in 0.1 M Tris buffer (pH 7.8). After 1 hour, the retina was washed with water, stained via conventional hematoxylin and eosin (H&E) methods, and observed via light microscopy. The total number of pericytes and acellular capillaries were counted in 5 randomly selected areas (1 mm x 1 mm) within the retinal capillaries, and the former was presented as a ratio relative to normal control mice. Pericytes were identified based on their morphology (small, spherical, and densely stained) and their location on capillary walls. Acellular capillaries were distinguished by the absence of oval or elongated retinal endothelial cell nuclei within their vessel walls.

### FITC-dextran staining

To visualize vascular leakage, 50 mg/mL of FITC-dextran (FD2000S; Sigma-Aldrich) was administered via tail vein injection. After 30 minutes, the mice were sacrificed and the enucleated eyeballs were fixed in 10% formalin solution for 1 hour before washing with PBS. The retina was isolated from the RPE-choroid complex and four equidistant cuts made to generate flat mounts prior to observation via fluorescence microscopy (Eclipse Ti-U; Nikon, Tokyo, Japan) at 50X magnification, with ImageJ used to determine FITC intensity.

### Determination of retinal cell layer thinning and TUNEL assay

H&E staining was performed on frozen transverse sections, including the optic nerve head, before using ImageJ to visualize the cell layers. Changes of the inner retina from the nerve fiber layer to the inner nuclear layer were calculated to determine, as a ratio, the extent to which retinal cell layer thinning occurred. This ratio is defined as: [mean(ratio of inner retina) = (thickness from nerve fiber layer to inner nuclear layer) / (thickness from nerve fiber layer to outer nuclear layer)]. The manufacturer’s protocol was followed to perform the TUNEL assay (12156792910; Roche Diagnostics), after which the frozen sections were washed 3 times in PBST for 10 minutes apiece and stained with DAPI for cell nuclei visualization purposes.

### Statistical analysis

One-way ANOVA testing was used for statistical analysis, with significant difference determined at *: p < 0.05; **: p < 0.01; or ***: p < 0.001. As at least 5 data points were needed to determine significance, the sample size in this study was 5 mice. Dot plot graphs were used to visualize the data and include significance and mean standard error of mean values. These procedures were done using GraphPad Prism (GraphPad Software, San Diego, CA). Among the animals used in this study, mice that did not successfully generate the diabetic mouse model and those sacrificed 1 month post-intravitreal injection were excluded from analysis. All image files are available from the Harvard Dataverse database (https://dataverse.harvard.edu/dataset.xhtml?persistentId=doi:10.7910/DVN/NXOYYG).

## Results

### In vivo efficacy of rAAV2-shmTOR-SD

**Fig 1:**
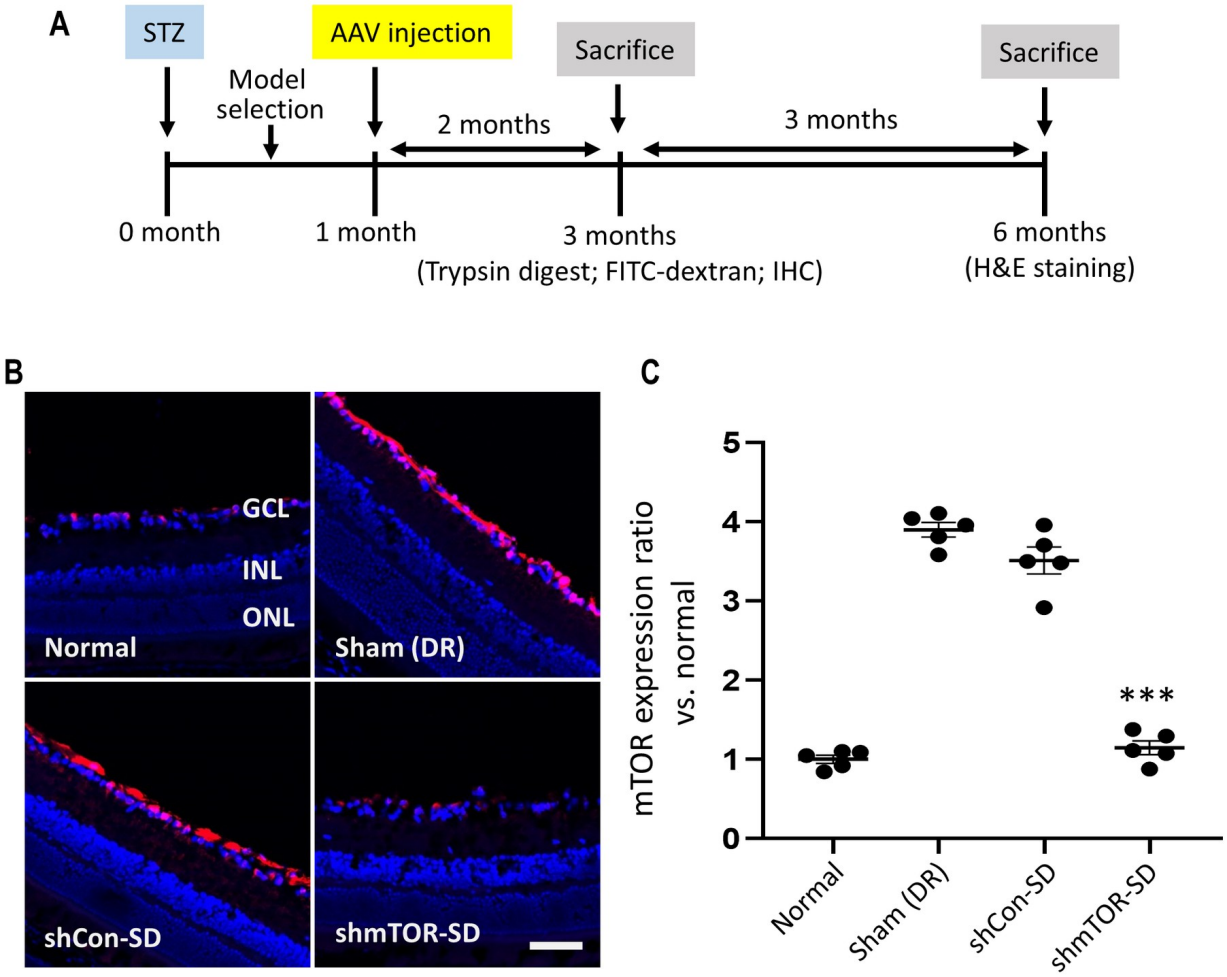
In vivo characterization of rAAV2-shmTOR-SD. (a) Experimental schematic. (b) Immunohistochemistry performed on transverse retinal sections sampled two months post-intravitreal injection showed that the therapeutic virus vector is able to effect the long-term inhibition of mTOR, which was elevated in control mice and those injected with rAAV2-shCon-SD. Scale bar = 50 μm. (c) Quantification of mTOR expression levels, relative to normal mice. GCL, ganglion cell layer; INL, inner nuclear layer; ONL, outer nuclear layer. Data represented as mean ± SEM (n = 5).

After establishing the STZ-induced diabetic mouse model (Fig 1A), the in vivo activity of the therapeutic virus vector in inhibiting mTOR was compared to three control groups: sham-treated mice in which DR had been induced via STZ, mice administered both STZ and a control shRNA-containing virus vector, and normal mice naïve to STZ treatment (Fig 1B). Relative to the latter group, anti-mTOR immunostaining performed on mouse retinal sections sampled 2 months post-intravitreal injection showed that mTOR expression was significantly upregulated in the sham-treated (3.898 ± 0.209) and rAAV2-shCon-SD-administered (3.510 ± 0.385) control groups, whereas rAAV2-shmTOR-SD treatment (1.146 ± 0.197; p < 0.001) resulted in mTOR levels comparable to that of normal control mice (1.000 ± 0.114) (Fig 1C). This demonstrates that the therapeutic virus vector was able to effectively transduce mouse retinas and therein effect long-term mTOR inhibition.

### Retinal pericyte loss, acellular capillary formation, and vascular leakage are reduced by rAAV2-shmTOR-SD

**Fig 2:**
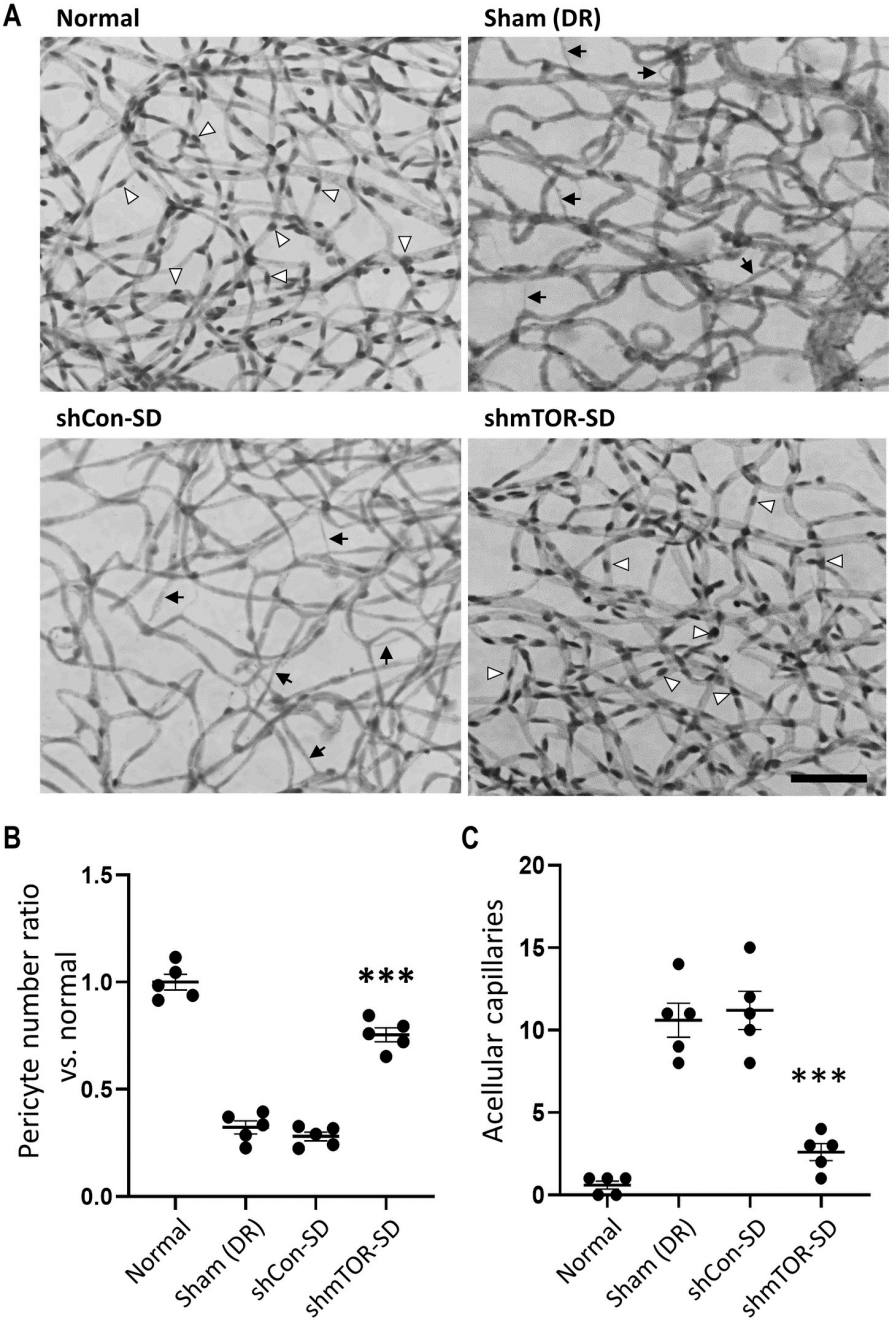
rAAV2-shmTOR-SD reduces retinal pericyte loss. (a) rAAV2-shmTOR-SD administration was shown to protect the diabetic retina against (b) pericyte loss, visualized via white arrowheads, and (c) acellular capillary formation, shown using black arrows, which lead to DR-related vasodegeneration. This early DR process was not observed in normal mice but readily detectable in mice treated with rAAV2-shCon-SD and sham-treated control mice. Scale bar = 50 μm. Data represented as mean ± SEM (n = 5).

Among the earliest manifestations of DR pathology [4], the loss of pericytes (Fig 2A, white arrowheads) was readily observed in sham- (0.320 ± 0.067) and control virus vector-treated (0.280 ± 0.045) mice, whereas it was markedly reduced by rAAV2-shmTOR-SD treatment (0.750 ± 0.072; p < 0.001). The occurrence of this key histopathological characteristic of DR [27] was determined 3 months after establishing the animal model and 2 months after intravitreal administration of the virus vectors by quantitating the number of pericytes (Fig 2B) in the mouse retinas relative to the normal control group (1.000 ± 0.082).

Pericyte loss can lead to the development of retinal non-perfusion [4, 12] and is visualized in trypsin digest preparations of diabetic models as acellular capillaries [12] (Fig 2A, black arrows), which were generally absent in normal control mice (0.600 ± 0.548). However, they were found in the STZ-induced diabetic mouse model (10.600 ± 2.302) and the control shRNA-treated control group (11.200 ± 2.588), while injection with the therapeutic virus vector (2.600 ± 1.140; p < 0.001) was shown to significantly protect against the formation of acellular capillaries (Fig 2C).

**Fig 3:**
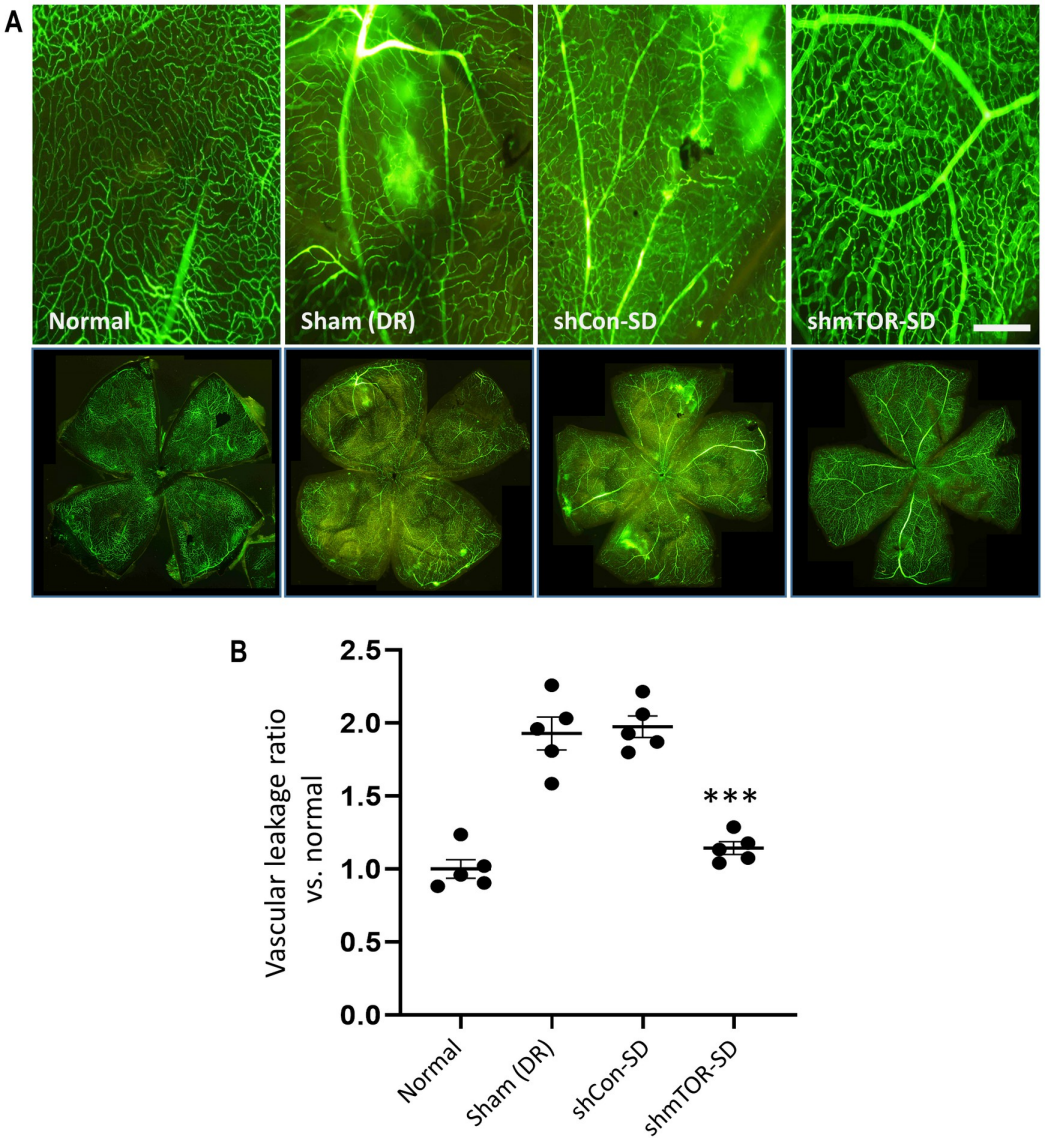
Vascular permeability visualized via FITC-dextran staining. (a) Significant amounts of retinal vessel leakage, appearing as indistinct regions of blurred staining, can be observed throughout the retinas of the sham-treated STZ-induced diabetic mouse models and those administered with the virus vector containing a control shRNA, whereas rAAV2-shmTOR-SD injection resulted in a marked decrease in vascular permeability. Scale bar = 50 μm. (b) Vascular leakage levels were determined from 5 randomly sampled areas of the retinal flat mounts, and the quantifications normalized relative to mice not treated with STZ. Data represented as mean ± SEM (n = 5).

Retinal non-perfusion is associated with vasodegeneration [12, 28] and the development of leaky vessels, another hallmark of DR. FITC-dextran staining of retinal flat mounts (Fig 3A), performed 2 months post-intravitreal injection and normalized to mice not treated with STZ (1.000 ± 0.142), showed that vascular permeability was markedly suppressed by rAAV2-shmTOR-SD administration (1.143 ± 0.096; p < 0.001) to levels comparable to the normal control group. On the other hand, elevated levels of vascular leakage were observable in the retinas (Fig 3B) of sham- (1.928 ± 0.252) and rAAV2-shCon-SD-treated (1.974 ± 0.165) mice, a process which, like pericyte loss, contributes to DR pathophysiology.

### rAAV2-shmTOR-SD protects against retinal cell layer thinning and is anti-apoptotic

**Fig 4:**
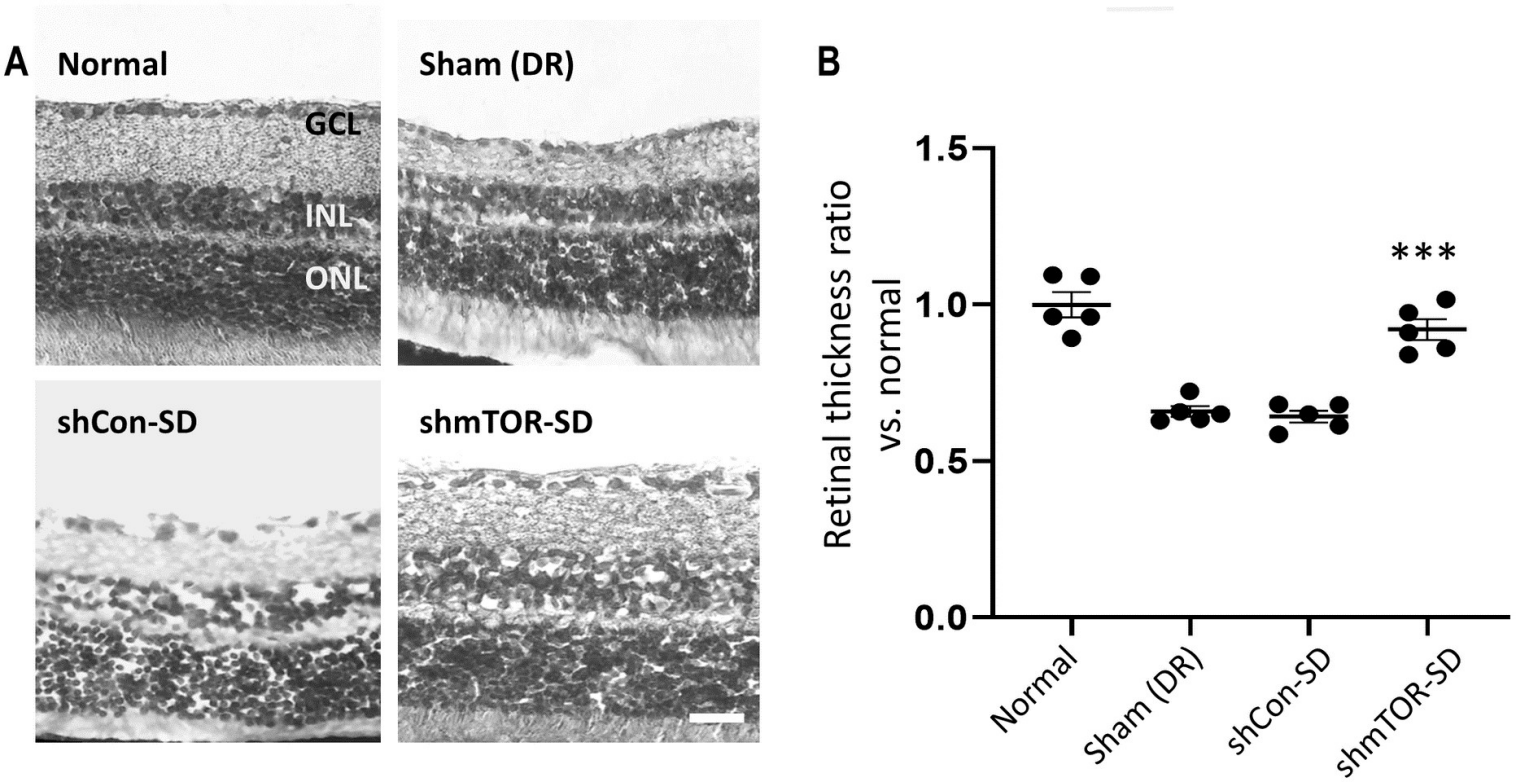
Visualization of retinal cell layers. (a) H&E staining was used to demonstrate that rAAV2-shmTOR-SD significantly reduced retinal cell layer thinning, which occurred in mice administered with a control shRNA-containing virus vector as well as sham-treated mice, suggesting that the therapeutic virus vector may possess neuroprotective properties. Scale bar = 50 μm. (b) Retinal cell layer thickness ratios normalized relative to normal control mice. Data represented as mean ± SEM (n = 5).

Relative to the normal control group (1.000 ± 0.089), sham-treated animals (0.659 ± 0.038) and mice administered with the control shRNA (0.642 ± 0.042) exhibited significant thinning of their retinal cell layers. Among the affected areas were the inner nuclear layer and the ganglion cell layer (Fig 4A), the latter being particularly susceptible to neurodegenerative effects [29]. On the other hand, rAAV2-shmTOR-SD treatment (0.920 ± 0.074; p < 0.001) resulted in markedly reduced retinal cell layer thinning for up to 5 months post-administration (Fig 4B).

**Fig 5:**
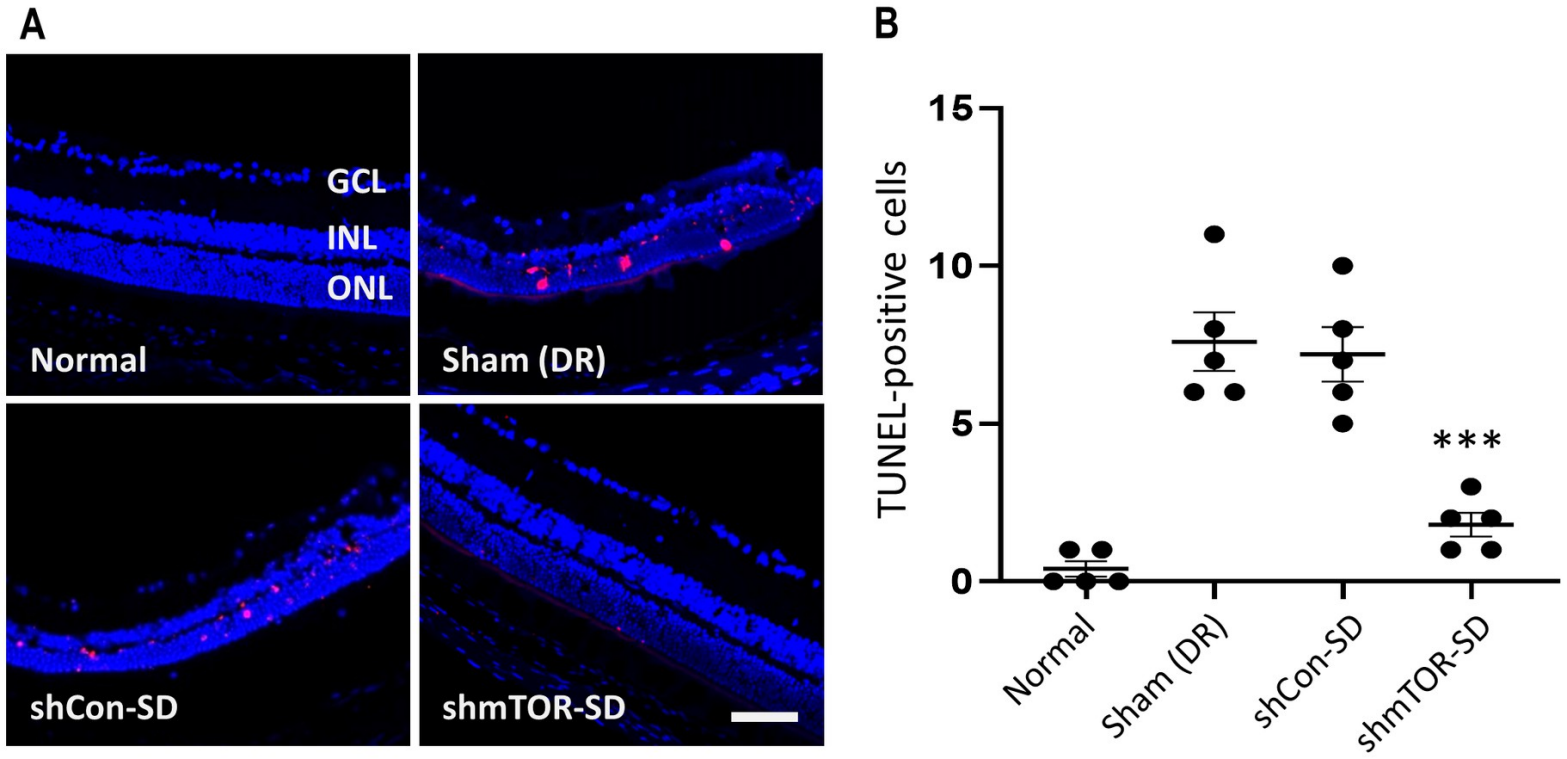
Anti-apoptotic effect of rAAV2-shmTOR-SD, as determined via TUNEL assay. (a) TUNEL-positive cells could not be found in transverse retinal sections of the normal control group, which were not treated with STZ, but were detected in sham-treated control mice and those injected with rAAV2-shCon-SD. Significantly fewer apoptotic cells were observed in mice treated with the therapeutic virus vector. Scale bar = 50 μm. (b) Number of TUNEL-positive cells. Data represented as mean ± SEM (n = 5).

Throughout its development, the mTOR-inhibiting shRNA has been shown to possess anti-apoptotic qualities [19, 22, 24], which was confirmed here in the STZ-induced diabetic mouse model. TUNEL-positive cells were found speckled throughout the retinal cell layers (Fig 5A) of the sham-control group (7.600 ± 2.074) and those administered with rAAV2-shCon-SD (7.200 ± 1.924). Meanwhile, apoptotic cells were largely absent from normal control animals (0.400 ± 0.548) and mice injected with the therapeutic virus vector (1.800 ± 0.837; p < 0.001), when measured 2 months post-administration (Fig 5B).

### Effects of rAAV2-shmTOR-SD on retinal cells

**Fig 6:**
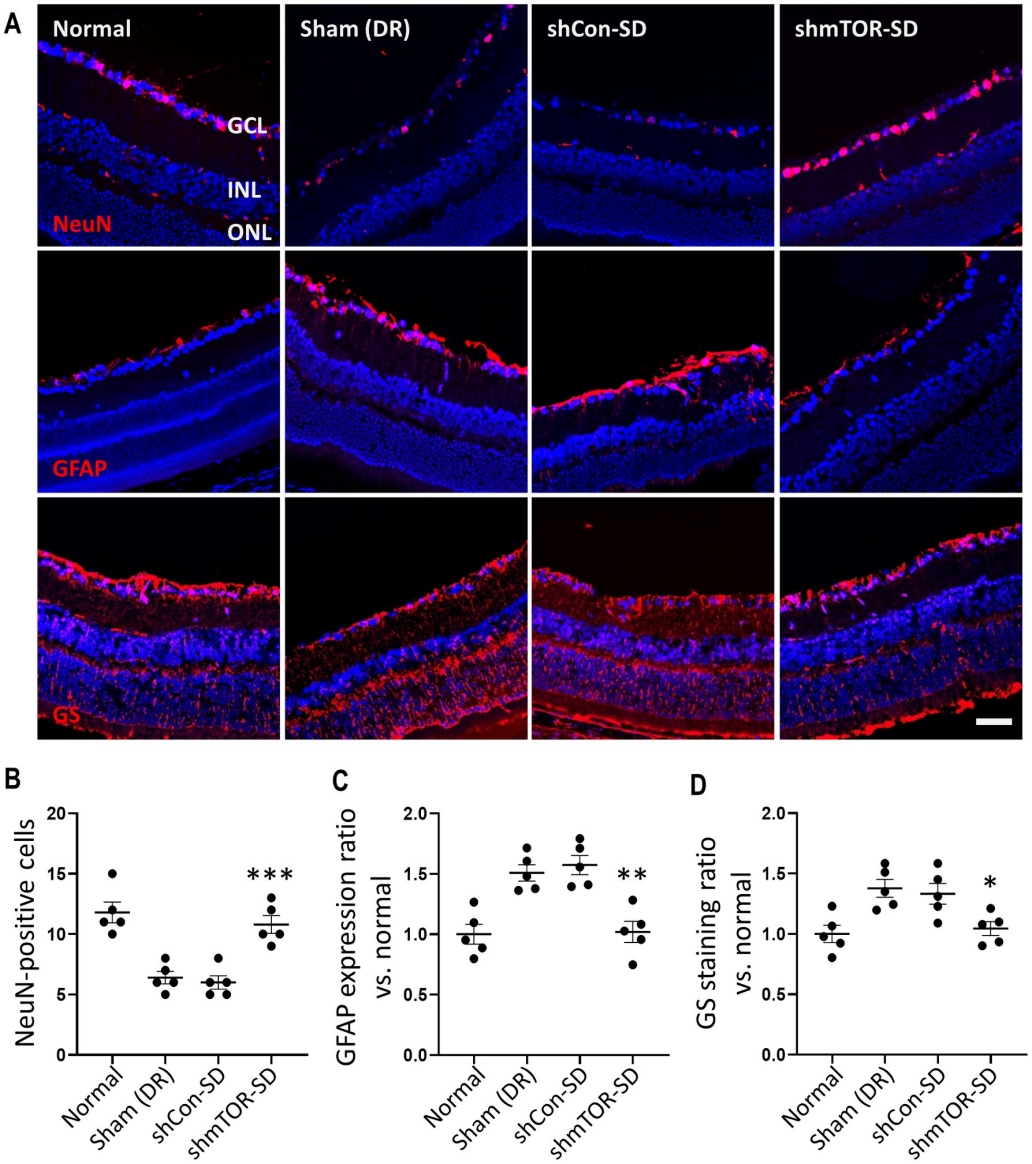
rAAV2-shmTOR-SD activity in various retinal tissues. (a) Frozen section samples were stained with anti-NeuN, anti-GFAP, or anti-GS to visualize the ganglion cell layer, glial cells, and activated Müller cells in the retinas of the STZ-induced mouse model. Two months post-intravitreal injection, it was seen that rAAV2-shmTOR-SD administration led to significant decreases in Müller cell activity and ganglion cell reduction, both of which were readily observed in animals treated with rAAV2-shCon-SD, as well as the sham-treated control group. Scale bar = 50 μm. (b) Determination of the number of NeuN-positive cells, alongside quantification of (c) GFAP and (d) GS expression as ratios relative to normal control rats. Data represented as mean ± SEM (n = 5).

Having been shown to transduce the retinas of STZ-induced diabetic mice to directly inhibit mTOR, as well as reduce cell death therein, immunohistochemistry was used to determine the effects of the therapeutic virus vector on various retinal cell types. Anti-NeuN, anti-GFAP, and anti-GS were used to stain the ganglion cell layer, glial cells, and activated Müller cells, respectively. As can be seen (Fig 6A), rAAV2-shmTOR-SD administration yielded results similar to the normal control group, whereas rAAV2-shCon-SD treatment and sham-treated mice correlated to one another (Table 1). rAAV2-shmTOR-SD treatment mitigated the loss of ganglion cells (Fig 6B) while leading to a reduction of glial cell activity (Fig 6C), Müller cells (Fig 6D) in particular, further suggesting that it may be neuroprotective.

**Table 1:** Retinal tissue tropism.

GROUP	NeuN	GFAP	GS
	(positive cell num.)	(vs. normal)	(vs. normal)
**Normal**	11.800 ± 1.924	1.000 ± 0.184	1.000 ± 0.160
**Sham (DR)**	6.400 ± 1.140	1.508 ± 0.151	1.378 ± 0.167
**rAAV2-shCon-SD**	6.000 ± 1.225	1.573 ± 0.178	1.332 ± 0.192
**rAAV2-shmTOR-SD**	10.800 ± 1.643	1.019 ± 0.194	1.045 ± 0.127
**p-value**	< 0.001	< 0.01	< 0.05

## Discussion

Here, we explored in a STZ-induced diabetic mouse model the extent to which direct mTOR inhibition was able to address various aspects of DR. Intravitreally-injected rAAV2-shmTOR-SD downregulated mTOR expression in the mouse retinas while having an effect on tissues crucial to the pathophysiology of DR. It was able to reduce retinal cell layer thinning while exerting an anti-apoptotic effect, suggesting that the therapeutic virus vector may be neuroprotective. rAAV2-shmTOR-SD was additionally found to protect against pericyte loss, the formation of acellular capillaries, and vascular leakage, which play major roles in DR progression.

As a frequently occurring complication of DM, DR is influenced by aspects of the more general disorder, hyperglycemia, for example, which has been linked to pericyte loss in the diabetic retina [4]. An influential factor in DR progression [12], pericyte loss was shown to be markedly reduced by rAAV2-shmTOR-SD (Fig 2). The loss of pericytes, which are important to normal retinal function [27] and play a crucial role in maintaining the structural integrity of retinal vessels [12], contribute to the development of areas of non-perfusion and other vessel abnormalities, including capillary occlusion and/or ischemia [4]. The hypoxic conditions thereby produced in the retinal environment lead to the activation of hypoxia inducible factor 1 (HIF-1) and the subsequent upregulation of VEGF, the key driver of PDR-related NV and a major contributor to DR progression overall. With pericytes linked to proper tight junction function [12] and several studies suggesting that VEGF may induce tight junction conformational changes [5], dysfunctions of the former and increases in the latter may result in the development of vascular leakage, a characteristic of DR which FITC-dextran staining showed was significantly reduced by rAAV2-shmTOR-SD (Fig 3).

Inextricably associated with diabetes mellitus (DM), inflammation exerts a significant influence over the pathophysiology of DR, demonstrated by the variety of anti-inflammatory approaches under investigation as potential DR treatments [30]. Müller cells are especially involved in DR-associated inflammatory processes, as hyperglycemia induces Müller cells to express a number of pro-inflammatory cytokines [4], including IL-1β and TNF-α, which together upregulate IL-8 [30]. Müller cells are additionally a major source of VEGF in DR [31], which, besides being the most potent driver of neovascularization during PDR, is also pro-inflammatory [5, 32] and increases vascular permeability [5]. Due to the multifarious effects Müller cells have DR progression, they are a major cellular target of rAAV2-shmTOR-SD, the administration of which led to reduced Müller cell activity, as shown by GS staining (Fig 6). This aspect is crucial to the therapeutic efficacy of the virus vector, as hyperglycemia has been observed to lead to the proliferation of Müller cells [33] and the subsequent potential creation of a positive feedback loop wherein the DR-associated activities of Müller cells become amplified.

Inflammation has also been linked with the induction of apoptosis in DR [27], which is implicated with the development of leaky vessels [4, 27] and retinal neurodegeneration [3, 29]. The latter affects the neurovascular unit (NVU) [3, 4, 31], which consists of pericytes, endothelial cells, ganglion cells, and glial cells, among others [2, 3]. Noted for the loss of retinal ganglion cells and multiple amacrine cell types [3], as well as a thinning of the inner nerve fiber layer [29], neurodegeneration is considered to be an important component of DR pathology [34, 35]. The ability of the therapeutic virus vector to reduce the extent to which retinal cell layer thinning occurred, as determined by calculating the changing thickness of the inner retina, which includes the inner nerve fiber layer and ganglion cell layer (Fig 4), while also reducing apoptotic activity (Fig 5), the cell death mechanism by which neurodegeneration occurs [27, 34, 35], suggests that it may be neuroprotective. This was supported by anti-NeuN staining, which demonstrated that rAAV2-shmTOR-SD administration was associated with a reduction in retinal ganglion cell loss. Further support for the neuroprotective qualities of the therapeutic virus vector was provided by reduced levels of anti-GFAP and anti-GS staining (Fig 6), as glial cell activation and dysfunction, characterized by increased GFAP expression [36] and glutamate synthesis by Müller cells [3], has also come to be seen as a key feature of DR-related retinal neurodegeneration [35].

As shown, DR pathophysiology is influenced by a variety of processes, meaning that inhibiting a single pathway, such as neovascularization, may be inadequate as a therapeutic modality. Therefore, a more comprehensive treatment strategy with broad effects may be preferred, even beyond various combination therapies, for which mTOR inhibition demonstrates great promise. mTOR exists as a constituent component of mTOR complex 1 (mTORC1), mTOR complex 2 (mTORC2) [13], and the recently identified mTOR complex 3 (mTORC3) [37], which together modulate a great number of downstream signaling pathways [13]. Rapamycin and its structural analogues, called rapalogs, have previously been explored as potential treatments for DR [15, 38] due to their anti-angiogenic qualities. These first-generation mTOR inhibitors target mTORC1 [13] and canonically reduce neovascularization by acting through HIF-1α, its downstream target, to downregulate VEGF [39]. However, VEGF is also a target gene of the mTORC2-regulated HIF-2α [40], and while second-generation mTOR inhibitors, which block the phosphorylation of all downstream targets of mTOR, have demonstrated potential as a DR treatment [14] by downregulating mTORC1 and mTORC2 [13], reactivation of the complexes eventually occurs via various mechanisms [13, 19, 41]. As such, the effectiveness of these mTOR-inhibiting agents in providing sustained VEGF suppression is limited [13]. Combined with the almost wholly unexplored nature of mTORC3 and the possibility that other possible mTOR complexes may exist, RNAi may prove to be an effective method to provide direct mTOR inhibition.

**Fig 7:**
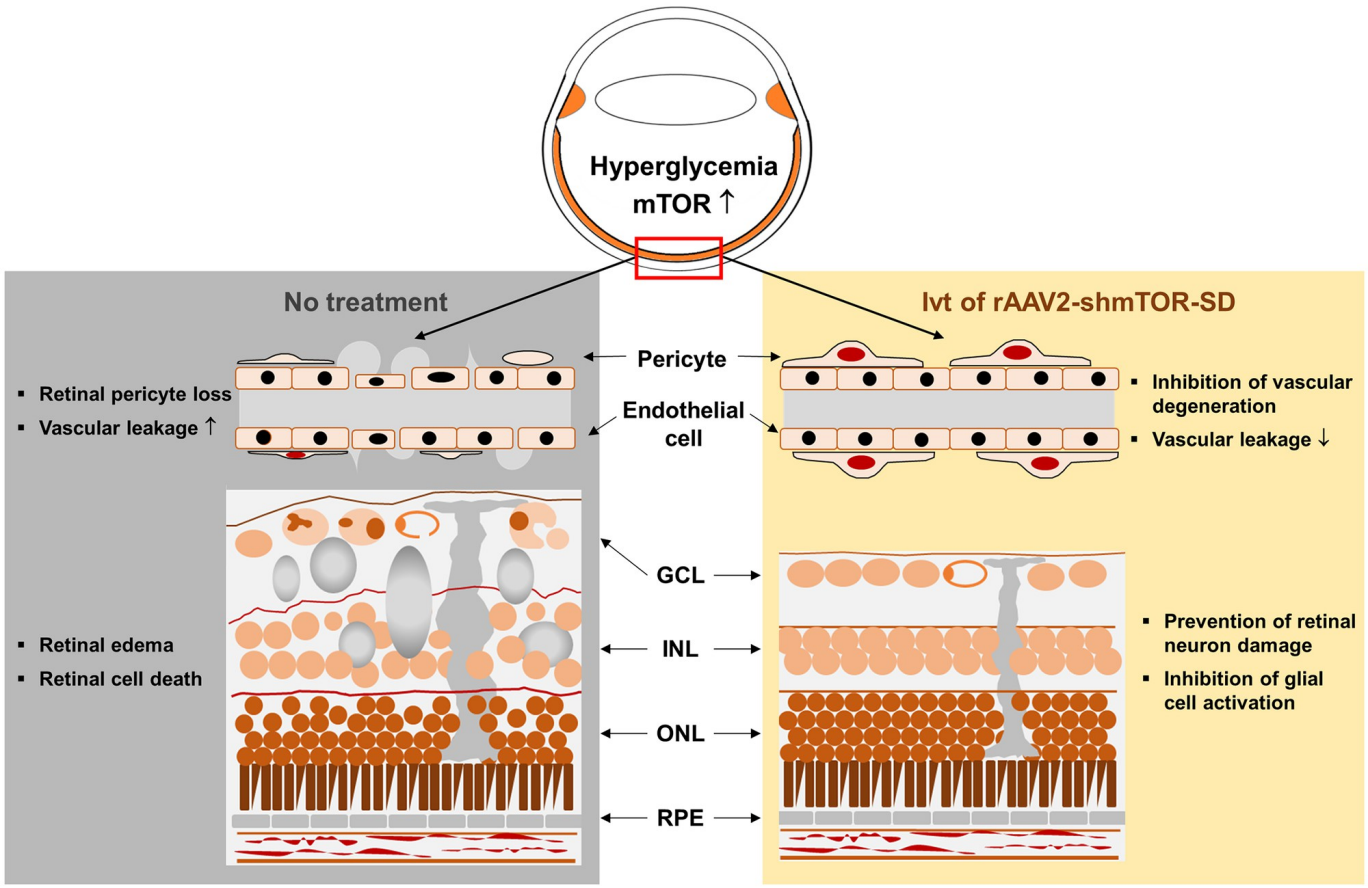
Therapeutic efficacy of a novel mTOR-inhibiting shRNA for the treatment of diabetic retinopathy. Early DR processes include pericyte loss and vascular leakage, which may lead to the development of edema, cell death, and disruptions in the retinal cell layers if left untreated. Intravitreal administration of rAAV2-shmTOR-SD was shown to effectively address these aspects of DR in a STZ-induced diabetic mouse model, further demonstrating its potential as a human gene therapeutic.

Although DR-associated pathologic retinal proliferation is not recapitulated in STZ-induced diabetic mice [42], early processes acting in concert to drive DR pathoprogression are observable [23], demonstrating its utility as a model system for studying DR pathogenesis and development. Under the influence of readily-inducible hyperglycemia [42–44], these processes include the loss of pericytes and subsequent retinal vessel dysfunction [23], chronic inflammation [45], and neurodegeneration [29]. Here, we were able to show that rAAV2-shmTOR-SD effectively transduces the retinas of the STZ-induced mouse model (Fig 1) to directly downregulate mTOR therein. By exerting therapeutically relevant effects in its cellular targets (Fig 6), including Müller cells and the ganglion cell layer, rAAV2-shmTOR-SD also protected against the thinning of retinal cell layers (Fig 4) while suppressing apoptosis (Fig 5). It was additionally shown to protect against pericyte loss and acellular capillary formation (Fig 2), whose consequence include vascular leakage, a process also reduced by rAAV2-shmTOR-SD (Fig 3). These results (Fig 7), combined with our previous work examining the ability of the mTOR-inhibiting shRNA to reduce neovascularization and exert an anti-inflammatory effect [19], show that rAAV2-shmTOR-SD has an effect on multiple aspects of DR pathophysiology, thereby continuing to demonstrate its promise as a gene therapeutic strategy versus the condition.

## Supporting information

S1 Table Body weight (g) of STZ-induced diabetic mice. (XLSX)

S2 Table Blood glucose (mg/dL) of STZ-induced diabetic mice. (XLSX)

S1 Fig mTOR inhibition by rAAV2-shmTOR-SD in normal control mice not treated with streptozotocin. (TIF)

S2 Fig Secondary antibody-only immunohistochemistry for the visualization of possible non-specific binding. (TIF)

S1 Data (XLSX)
