# Supplementary figures and images for: mTOR inhibition as a novel gene therapeutic strategy for diabetic retinopathy
Source: PLoS One. 2022 Jun 16;17(6):e0269951. doi: 10.1371/journal.pone.0269951 (PMC9202865; doi:10.1371/journal.pone.0269951)

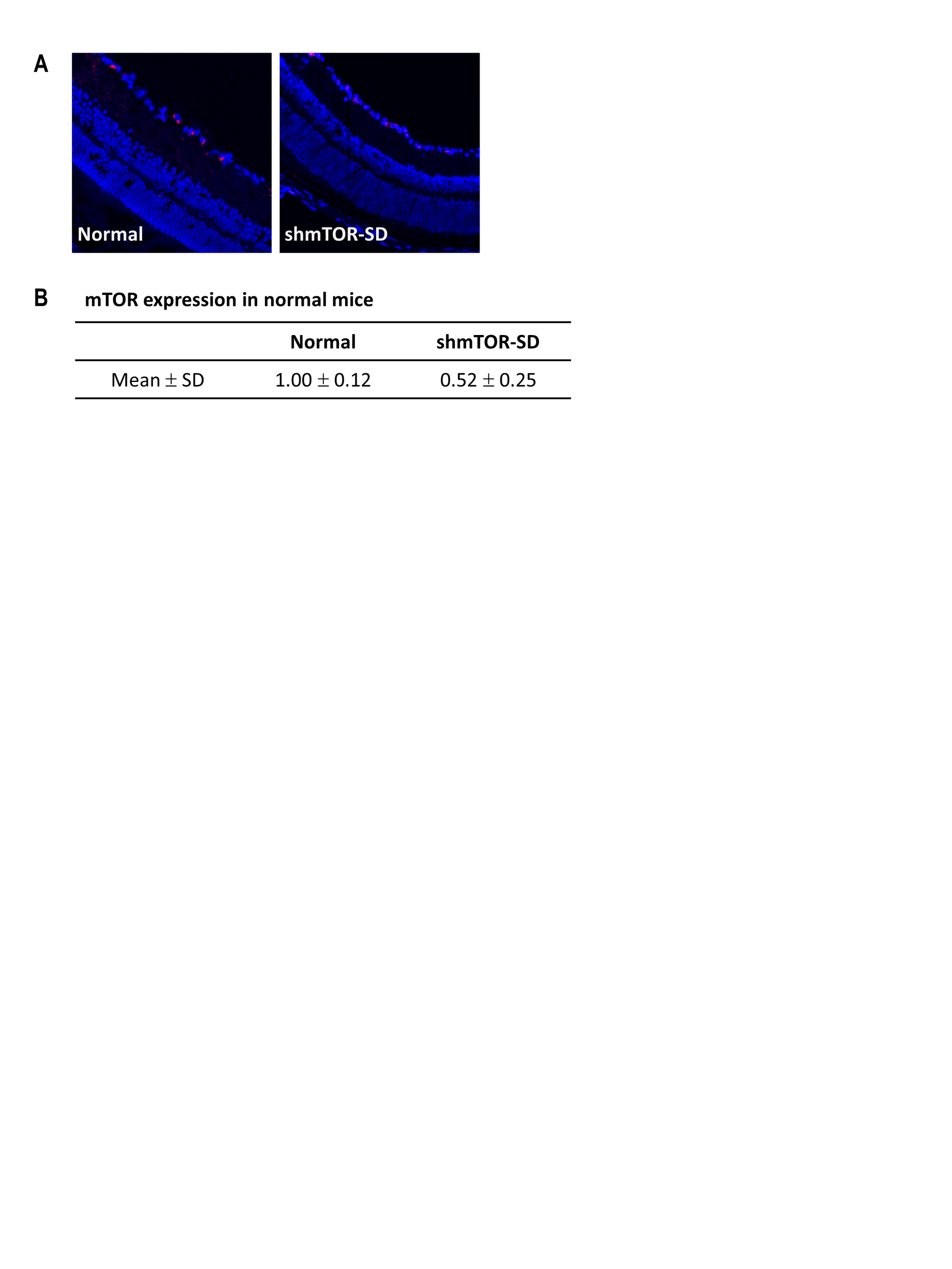

Supplement: S1 Fig — (TIF) [file pone.0269951.s003.tif]

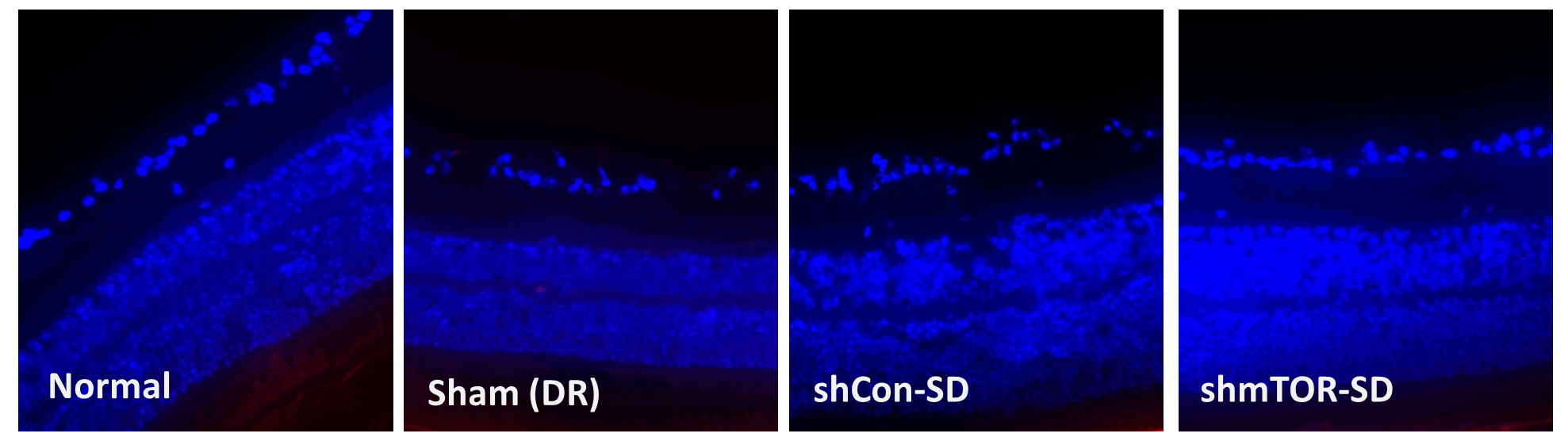

Supplement: S2 Fig — (TIF) [file pone.0269951.s004.tif]
